# Hierarchical Brain Networks Active in Approach and Avoidance Goal Pursuit

**DOI:** 10.3389/fnhum.2013.00284

**Published:** 2013-06-17

**Authors:** Jeffrey M. Spielberg, Wendy Heller, Gregory A. Miller

**Affiliations:** ^1^Institute for Personality and Social Research, University of California Berkeley, Berkeley, CA, USA; ^2^Department of Psychology, University of Illinois at Urbana-Champaign, Champaign, IL, USA; ^3^Department of Psychology, University of Delaware, Newark, DE, USA; ^4^Department of Psychology, University of California, Los Angeles, CA, USA

**Keywords:** goal pursuit, approach motivation, avoidance motivation, executive function, prefrontal cortex, laterality, abstraction gradient

## Abstract

Effective approach/avoidance goal pursuit is critical for attaining long-term health and well-being. Research on the neural correlates of key goal-pursuit processes (e.g., motivation) has long been of interest, with lateralization in prefrontal cortex being a particularly fruitful target of investigation. However, this literature has often been limited by a lack of spatial specificity and has not delineated the precise aspects of approach/avoidance motivation involved. Additionally, the relationships among brain regions (i.e., network connectivity) vital to goal-pursuit remain largely unexplored. Specificity in location, process, and network relationship is vital for moving beyond gross characterizations of function and identifying the precise cortical mechanisms involved in motivation. The present paper integrates research using more spatially specific methodologies (e.g., functional magnetic resonance imaging) with the rich psychological literature on approach/avoidance to propose an integrative network model that takes advantage of the strengths of each of these literatures.

*If you want to live a happy life, tie it to a goal, not to people or things*.

—Albert Einstein

## Approach and Avoidance Goal Pursuit

The ability to pursue goals effectively is critical for attaining long-term health and well-being. In fact, independently meeting even our most basic needs requires some degree of successful goal pursuit. Goal pursuit can be defined as “responses intentionally performed to bring about or maintain a desired state” or “responses intentionally performed to control or prevent an undesired state,” often termed approach and avoidance goal pursuit, respectively (Moskowitz and Grant, 2009). Research suggests that this approach/avoidance dichotomy is a central characteristic of goal pursuit and corresponds to two fundamental motivational systems (Elliot, 2006).

An extant body of neuroscience research provides a valuable foundation for understanding approach/avoidance functions. In particular, findings suggest that the prefrontal cortex (PFC) is lateralized with respect to motivational direction, with left PFC associated with approach and right PFC with avoidance (for review, see Spielberg et al., 2008). However, a number of factors limit the utility of this work (Miller et al., 2013). For example, research investigating approach/avoidance lateralization in PFC often fails to elucidate the specific aspects of approach/avoidance thought to be associated with prefrontal asymmetry (Tomarken and Zald, 2009). More broadly, neuroscience research on approach/avoidance goal pursuit and motivation has largely failed to take advantage of the rich psychological literature in this area, limiting the extent to which neural mechanisms involved in more complex goal-pursuit processes can be represented. Therefore, integration of the literature on goal pursuit in (lateralized) PFC with nuanced conceptualizations of approach/avoidance that deconstruct these fairly broad constructs into component processes will be necessary to achieve a full understanding of the neural mechanisms of goal pursuit.

Historically, an additional limiting factor in investigations of approach/avoidance lateralization in PFC has been a reliance on low-density electroencephalography (EEG), which does not allow for very precise spatial localization of activity (Tomarken and Zald, 2009). In the last two decades, functional magnetic resonance imaging (fMRI) has come to the fore as the dominant method to attain spatial specificity (although lateralization is often ignored or assessed inadequately; Herrington et al., 2005). However, the quest for spatial specificity has had an unfortunate consequence. Specifically, the vast majority of fMRI research has relied on what has been termed the “modular paradigm,” in which processes are mapped simplistically onto specific brain regions, and connectivity among regions is not taken into consideration (Miller, 2010; Meehan and Bressler, 2012). Given that regions of the brain are nodes in highly interconnected networks rather than independent “islands” (Sporns, 2011), and changes in connectivity have a distributed impact throughout a network (Levit-Binnun and Golland, 2011), attempts to represent the neural mechanisms of goal pursuit and motivation will necessarily be limited until connectivity between network nodes is taken into account. Fortuitously, a recent explosion of methods for network identification and description (Bressler and Menon, 2010) has given us the tools to dramatically increase the complexity of the processes that we are able to represent, moving us closer to accurate representations of our constructs of interest (Chen and Berrios, 1998).

The present paper attempts to fill these gaps in the literature by presenting a network model of the neural instantiation of approach/avoidance goal pursuit. It reviews and integrates nuanced psychological models of approach/avoidance into a framework for understanding the extant neuroscience research, presents a preliminary structure of network connections, and outlines the way in which network nodes may interact to pursue goals. Importantly, this model incorporates hemispheric laterality, often ignored in fMRI research. Given the focus on cortical lateralization, the scope of the present paper is limited largely to PFC.

## Key Goal-Pursuit Processes

As mentioned above, what have been termed the approach and avoidance motivational systems form the basis for the two types of goal pursuit. Although many definitions of motivation have been proposed, several functional aspects are fairly consistent. Specifically, many theories conceptualize motivation as internal processes that select goals based on their predicted value (e.g., reward or punishment), initiate behavior to achieve goals, and maintain goal-directed action (e.g., Jones, 1955; Lindsley, 1957; Campbell and Pritchard, 1976). Thus, motivation is necessary for an organism to pursue goals.

However, the construct of motivation does not encompass all processes needed to pursue goals. Many theorists have proposed that cognition interacts with motivational processes during goal pursuit (e.g., Sorrentino and Higgins, 1986; Locke and Latham, 2002). Although usually not explicitly defined, cognition has often been conceptualized as “those processes that mediate the acquisition and representation of knowledge about the world” (Kuhl, 1986, p. 407), including skills and abilities (Locke, 2000). This work rests on the assumption that motivation and cognition are separable processes (Kruglanski, 1999). However, there does not appear to be sufficient grounds to assume this dichotomy, with many theorists asserting that motivation and cognition are, at the very least, highly overlapping and interdependent (Lazarus, 1991; Miller, 1996, 2010; Sherman and Sherman, 1999; Crocker et al., 2013), if not simply different facets of the same construct (Sorrentino and Higgins, 1986; Kruglanski, 1999).

Distinguishing between motivational and cognitive processes becomes even more difficult when considering executive function. Similar to cognition, the construct of executive function is often defined imprecisely and with a large amount of variability (Martin and Failows, 2010). At a broad level, executive function is often conceptualized as the processes by which goal-directed action is carried out (Banich, 2009). Therefore, executive function shares with motivation a fundamental focus on goal pursuit. However, these constructs appear to have separable aspects. For example, processes involved in the energization of behavior are often considered to be solely the province of motivation. Additionally, executive function is associated with abilities, such as shifting, updating, and inhibition (Miyake et al., 2000), that are not usually considered to be part of the construct of motivation. The present paper provides a model of how the psychological processes involved in the pursuit of goals are instantiated in the brain, rather than delineating those processes which belong to motivation vs. executive function (or cognition more generally). Given that the present paper builds on psychological models of motivation, a motivational framework will be privileged. However, some of the processes discussed in the present paper under the rubric of motivation could just as validly be conceptualized as cognitive or executive function.

## Hierarchical Approach and Avoidance Motivational Systems

Approach and avoidance motivation are hypothesized to form the “basic building blocks that underlie the complexity of human behavior” (Carver et al., 2000, p. 741). Several researchers have suggested that these motivational systems are comprised of a number of hierarchical levels, with lower levels of these models subservient to higher levels (Lang et al., 1998; Elliot, 2006; Scholer and Higgins, 2008). For instance, Higgins and colleagues (for a review, see Scholer and Higgins, 2008) proposed a structure with three levels: the system, strategic, and tactical levels. These levels are thought to be hierarchical, but the selection of approach and avoidance is independent at each level.

### System level

At the system level, approach and avoidance are defined in relation to the goal that is currently held. Specifically, the goal can be to approach a potential desirable outcome or avoid a potential undesirable outcome. The critical determinant at this level is how the individual views the goal-object (the outcome of focus), rather than the properties of the goal-object itself. Therefore, the same goal-object can be part of either an approach or avoidance goal, depending on the individual’s motivational orientation. Given two individuals striving to get an A on a test, one individual could view an A as an accomplishment that will bring them pleasure (a desirable outcome), whereas the other could view getting anything lower than an A as a failure that will bring them displeasure (an undesirable outcome). Based on their motivational orientation, the first individual wants to approach an A, whereas the second individual wants to avoid getting anything lower than an A.

Given that numerous conceptualizations of the goal construct are available in the literature, it is important to outline the specific operationalization used, in order to avoid confusion (Elliot and Fryer, 2008). Elliot and Niesta (2009, p. 58) suggested that a goal is defined as a “cognitive representation of a future object that the organism is committed to approach or avoid.” In this conceptualization, the goal construct includes a commitment to pursue the goal-object. This commitment, along with the representation of the object (e.g., stimulus properties, associated value), must be sustained over time. Thus, one function of the system level is to maintain the goal construct over time.

### Strategic level

**Figure 1:**
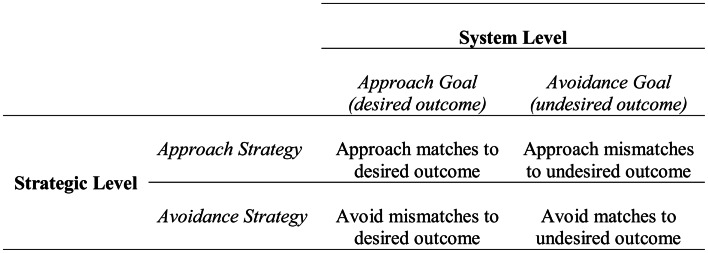
**Relationship between the system and strategic levels of the hierarchical model of motivation**. Figure adapted from Higgins et al. (1994).

At the strategic level, approach and avoidance are defined in relation to the means or process of attaining a potential desirable outcome or preventing a potential undesirable outcome. As shown in Figure 1, at the strategic level one can approach matches to a desirable outcome (i.e., outcomes consistent with the desired state) or mismatches to an undesirable outcome (i.e., outcomes inconsistent with the undesired state). Similarly, one can avoid mismatches to a desirable outcome (i.e., outcomes inconsistent with the desired state) or matches to an undesirable outcome (i.e., outcomes consistent with the undesired state). Therefore, when approaching a desirable outcome at the system level, one can either approach matches to that outcome or avoid mismatches to that outcome. For example, if the potential outcome was getting an A, approaching a match could be studying hard, and avoiding a mismatch could be staying away from situations that distract from studying. Similarly, when avoiding an undesirable outcome at the system level (e.g., not getting an A), one can either approach mismatches (e.g., studying hard) or avoid matches (e.g., avoiding distraction) to that outcome. Approach and avoidance at the strategic level reflect general/broad plans or means, rather than the specific instantiations of means, which are instead captured in the tactical level.

As discussed above, individuals commit to approaching or avoiding a certain end-state at the system level. In contrast, at the strategic level, a commitment can be made to the goal-pursuit plan, which Gollwitzer (1999) labeled an implementation intention (for reviews, see Gollwitzer, 1999; Parks-Stamm and Gollwitzer, 2009). Gollwitzer conceptualized implementation intentions as if-then plans that link goal-directed actions to anticipated opportunities to engage in these actions (i.e., committing to act in a certain way when a specific situation is encountered). For example, if a student commits to the goal of approaching an A, that student may form an approach plan to complete extra credit assignments (the action) whenever they are offered by the instructor (the situation).

Research indicates that forming implementation intentions increases the likelihood of goal attainment, especially for difficult-to-obtain goals (see Gollwitzer and Sheeran, 2006, for a meta-analysis of 94 studies indicating an effect size of *d* = 0.65). Gollwitzer (1999) attempted to explain this effect by suggesting that implementation intentions make the anticipated situation and planned response more cognitively accessible (i.e., primed). For instance, increasing the accessibility of the anticipated situation makes it easier to detect in the presence of distraction. Similarly, increasing the accessibility of the planned response makes it easier to select in the presence of competing responses. Research supports this hypothesis (for a review, see Gollwitzer et al., 2004), including in populations that experience executive dysfunction (e.g., children with Attention Deficit Hyperactivity Disorder, Gawrilow and Gollwitzer, 2008).

When implementation intentions are not formed, individuals must actively attend to the environment in order to detect opportunities to pursue the goal, which is more effortful and lacks the benefits discussed above (Gollwitzer, 1999). However, in some circumstances the formation of implementation intentions can be detrimental, and active monitoring may be the more successful choice. For example, implementation intentions can interfere with the pursuit of concurrent goals (Achtziger et al., 2012) unless the situation and action committed to in the implementation intention subserve both goals. Additionally, implementation intentions can be detrimental when novel goal-pursuit situations are encountered, because the heightened accessibility of the option committed to can direct attention away from novel situations (Parks-Stamm et al., 2007). In summary, at the strategic level, approach and avoidance goal-pursuit plans are selected and can either be committed to (i.e., the formation of implementation intentions) or actively managed.

### Tactical level

At the tactical level, approach and avoidance are defined in relation to the specific ways a strategy could be implemented in a particular context. For example, if the strategy were to study hard, an approach tactic could be setting aside a specific time to study. An avoidance tactic could be making sure that no important study materials are missing. The tactical level is still at a higher level than the actual behavior implemented in a given situation, because an avoidance tactic can be implemented through physically approaching a stimulus and vice versa. For example, an avoidance tactic, such as ensuring that no important study materials are missing, could be implemented with approach behavior, such as approaching a classmate to ask them to show you their study materials or approaching the professor to ask them whether your study materials are adequate.

Although the levels are considered to be independent, Scholer and Higgins (2008) hypothesized that individuals tend to be consistent across levels. Higgins (2000, 2005) accounted for this consistency by proposing that inconsistency across levels leads to disruption in motivational orientation (i.e., approach or avoidance), which, in turn, leads to decreases in goal engagement (i.e., the amount of attention and effort invested in the goal). When there is consistency across levels, motivational orientation is maintained, and goal engagement is sustained.

### Temperament level

Elliot (2006) proposed a complementary hierarchical model of approach/avoidance motivation. This model consists of a temperament level comprised of individual differences in sensitivity to desired (approach temperament) or undesired (avoidance temperament) potential outcomes and adoption of approach or avoidance goals accordingly (at the system level). For example, a student high on avoidance temperament faced with an upcoming test will be sensitive to the potential for failure (e.g., appearing unintelligent) and is likely to adopt the goal of getting an A on the test in order to avoid failure. In contrast, a student high on approach temperament will be sensitive to the potential for success (e.g., appearing intelligent) and is likely to adopt the goal of getting an A in order to approach success. These students share the same goal-object (i.e., getting an A), but the underlying reasons for that goal differ.

Along with sensitivity to particular outcomes, the temperament level also involves general tendencies to adopt approach and avoidance means to attain goals (likely at both the strategic and tactical levels). Thus, the temperament level should lead to a tendency to engage the approach and/or avoidance systems overall, providing another method by which consistency across levels is attained.

### Integrated model

In summary, combining Scholer and Higgins’ (2008) and Elliot’s (2006) models, the present proposal offers a hierarchical model of approach/avoidance motivation that consists of four levels: temperamental, system, strategic, and tactical. The temperamental level consists of broad tendencies to implement approach or avoidance goals and approach or avoidance means to attain these goals. The system level maintains the goals that are held, approaching desired outcomes and/or avoiding undesired outcomes. The strategic level represents the general means or process by which the goal will be pursued, and, at the tactical level, the strategy is instantiated in a specific context.

We propose that these levels can be conceptualized along a gradient of both abstraction and timescale, with higher levels being more abstract and typically having a longer timescale. For example, we posit that the system level has a longer time scale than the strategic level, because the goal must be maintained over time, during which a number of strategies can be employed. Thus, a goal must be maintained at the system level over longer periods than any particular strategy.

Similarly, we propose that the strategic level is more abstract than the tactical level, because the tactical level represents the implementation of the strategy in a given context. The system level is more abstract than the strategic level, because the same goal can be subserved by several strategies (i.e., equifinality, Martin and Tesser, 2009). Furthermore, goals themselves can range from concrete (e.g., get an A on a specific test) to abstract (e.g., be a nice person), whereas strategies are tied to specific goals. Thus, strategies can only be as abstract as the goals they are tied to. For example, it could be argued that a strategy such as “study hard” is more abstract than the goal of getting an A on a test. However, in this case, the strategy would be to study specific material (the material that will be tested) to the degree needed to attain an A in this particular class (which incorporates all the concrete information known about the class). If the strategy were simply to “study hard,” it would not be a strategy for this particular goal, but likely the larger goal of doing well at one’s studies overall. Thus, we suggest that, for a strategy to be tied to a particular goal, it must incorporate the parameters specific to that goal. This constrains the level of abstraction of a particular strategy, with the upper limit being the level of abstraction of the goal. Finally, the temperament level is the most abstract and has the longest time scale, as it reflects dispositions over the lifetime to activate a motivational orientation that is independent of specific goals.

The integrated hierarchical model proposed here implies that higher levels in the structure exert control over lower levels. For example, to attain a goal held at the system level, the goal must be maintained over time and appropriate strategies employed at appropriate times. Thus, processing at the strategic level is constrained by the particular goal held at the system level, and the system level must engage the strategic level when needed. Consequently, the role of the system level can be conceptualized as biasing processing in lower levels of the structure in support of an overarching goal. Given that multiple goals can be held at one time, the system level must also prioritize goals at any moment and bias processing accordingly. Therefore, one important function of this motivational system is to constrain processing at lower levels such that the organism continues to move toward the goal, accomplished through top-down biasing by levels higher in the hierarchy.

Although the proposed model is hierarchical, this is not to imply that communication between levels is always one-way (in fact, such a view is at odds with prefrontal neuroanatomy, e.g., Averbeck and Seo, 2008, as discussed below). Rather, bidirectional information flow between levels is vital to providing the feedback necessary to adaptively pursue goals (Fuster, 2008). In addition, the proposed hierarchy does not imply that inter-level communication must occur *only* through adjacent levels (e.g., the system level *must* bias the tactical level *only* through the strategic level). It appears likely that inter-level communication will occur in accordance with the hierarchy more often than not, because processing in adjacent levels will be more relevant that processing in non-adjacent levels. For example, information regarding the success of a particular tactic will have more utility for evaluating the larger strategy than for evaluating the larger goal (at the system level). However, information flow between non-adjacent levels is crucial in many cases. For example, given the complexity of our environment, the system level often maintains multiple goals simultaneously. In order to determine which particular goal to emphasize at any given moment, the tactical level must provide feedback to the system level about the estimated success of the tactics currently associated with each goal. Thus, the hierarchical nature of the proposed model still allows for the flexibility necessary to adaptively pursue goals.

The type of flexible communication described above is necessary in order to adaptively identify subgoals. That is, it is essential to set smaller, more proximal goals that comprise the steps necessary to attaining larger, distal goals (Kruglanski et al., 2002). For example, in order to obtain an academic degree, a student must complete a series of classes. Thus, completing each class is a subgoal necessary to the larger goal. An obvious question is *do subgoals differ from lower levels in the hierarchy*? We propose that these concepts, although similar, are relevant to different pieces of the process of pursuing longer-term goals. For example, a particular strategy is only relevant to the degree that it subserves the larger goal, whereas subgoals are themselves goals. That is, subgoals are committed to in and of themselves, which can become problematic when the subgoal becomes more important that the higher order goal it was meant to serve (D’Zurilla and Nezu, 2007). With relation to the proposed model, subgoals would be maintained by the system level once they were identified and committed to as goals. Thus, what may start out as a strategy/tactic becomes a subgoal once the individual identifies it as a discrete goal, apart from just a means to an end.

In conclusion, the integrated hierarchical model of approach/avoidance goal-pursuit proposed here is a nuanced conceptualization that deconstructs the fairly broad constructs of approach/avoidance into important component processes. We propose that this psychological model can serve as a framework for understanding the processes examined in neuroscience research on goal-pursuit systems.

## Neural Instantiation of Approach/Avoidance Goal Pursuit

Building on the proposed psychological model of approach and avoidance, we next attempt to integrate disparate bodies of neuroscience research to construct a preliminary model of the neural mechanisms instantiating the psychological model. In the present paper, we focus on PFC, given an overwhelming research base indicating that PFC is central to the pursuit of goals (Miller and Cohen, 2001; Fuster, 2008). Given that the approach/avoidance distinction is fundamental to the proposed model, we begin by reviewing research that has found such a distinction in prefrontal organization.

### Lateralization in prefrontal cortex associated with motivation

A long line of research suggests that PFC is lateralized with respect to motivational/emotional valence, with right PFC associated with avoidance motivation and unpleasant emotion, and left PFC associated with approach motivation and pleasant emotion (for review, see Heller et al., 2003; Miller et al., 2013). PFC lateralization with respect to motivational or emotional valence is supported by research using a number of different methodologies, including neuropsychological testing (e.g., Flor-Henry, 1976), brain lesion patients (e.g., Gainotti, 1972), and EEG (e.g., Davidson et al., 1990). Although PFC asymmetries have regularly been observed in EEG and other methodologies, they have been elusive in studies employing fMRI. This method complements EEG in that it provides better spatial resolution than traditional low-density EEG for locating specific areas of PFC involved in emotion and motivation. Herrington et al. (2005) were the first to demonstrate leftward lateralization for pleasant emotion using fMRI, which was localized to DLPFC. As discussed in Herrington et al. (2010), one reason why lateralization findings are uncommon in fMRI may be that lateralization is rarely tested directly. Indeed, region (including hemisphere) is almost never a factor or predictor in analyses of fMRI data. Statements are often made about what are in effect multiple simple-effects tests without a systematic evaluation of the underlying interaction.

### Motivation and executive function

Although motivation and executive function are both vital to goal-pursuit individually, we and others have argued that it is the interaction between these sets of processes that drives effective goal pursuit (e.g., Pessoa, 2009; Spielberg et al., 2011). Consider this scenario involving a student in her first year of college: it is the night before her first big test, and she planned to study for several hours and get a full night’s rest. However, her roommate, with whom she has yet to develop a relationship, has just invited her to a party taking place that evening. If the student is motivated more strongly to obtain an A on her test, she must inhibit distraction (i.e., engage executive function) caused by reminders of the party (e.g., seeing her roommate’s jacket) in order to fully concentrate on her material. However, if the student is motivated more strongly to build a friendship, she must inhibit distraction caused by reminders of her impending test (e.g., seeing a student from class at the party) in order to enjoy the party and fully interact with her roommate. In other words, what information is inhibited depends on which goal the student is motivated to pursue.

An emerging body of research consistently implicates areas of PFC in the integration of motivation and executive function processes (Gilbert and Fiez, 2004; Gray et al., 2005; Krawczyk et al., 2007; Locke and Braver, 2008; Rowe et al., 2008; Szatkowska et al., 2008). Such integration is consistent with conceptualizations of PFC as being necessary “to orchestrate thought and action in accordance with internal goals” (Miller and Cohen, 2001). For example, Pochon et al. (2002) examined the relation between reward processing, a facet of motivation, and performance on a working memory task. Results revealed that left DLPFC was activated by both working memory demands and increasing levels of reward. Taylor et al. (2004) conducted a similar study that examined the interaction between state motivation and working memory by manipulating motivation in terms of both reward and punishment. Consistent with the findings of Pochon et al. (2002), motivational processes interacted with working memory load in bilateral DLPFC. Several studies have also examined the interaction between motivation and inhibition-related processes. For example, Padmala and Pessoa (2010, 2011) found that monetary reward interacted with inhibition requirements in DLPFC (bilateral in one study, right only in the other study). In addition, Krebs et al. (2011) found that right DLPFC was activated when inhibiting goal-irrelevant reward associations.

Thus, several studies suggest that DLPFC is essential for the neural integration of motivation and executive function processes. These studies can be interpreted as manipulating the system level of the present hierarchical model of motivation, because they manipulate the reasons for the goal (i.e., to do well in order to obtain a reward or avoid a punishment). Thus, this research suggests that the system level is instantiated in (at least) DLPFC, which is consistent with research suggesting that DLPFC is involved in representing and maintaining goals (e.g., MacDonald et al., 2000). Although involvement of DLPFC was observed in all studies, there is inconsistency in the hemisphere which exhibited activation. Inconsistencies in the lateralization of DLPFC activation may be due several issues, including differences in the motivational manipulation used across the studies and the facet of executive function recruited. For example, Pochon et al. (2002) employed only a reward manipulation, consistent with the leftward lateralization found, whereas Taylor et al.’s (2004) motivational manipulation included both reward and punishment, consistent with the bilateral activation observed. However, all the studies recruiting inhibition used only reward and found right DLPFC activation (one study found bilateral activation), suggesting that task differences may play some part. The picture is further clouded by the fact that none of these studies actually tested laterality effects. Thus, the extent of the inconsistency is not clear.

### Motivational temperament and executive function

Extending the work on state motivation and executive function, recent research has examined the interaction of motivational temperament with executive function. For example, Spielberg et al. (2011) investigated moderation of neural activation associated with the color-word Stroop (1935) task by approach and avoidance temperament. Neural activation associated with incongruent words was contrasted with activation associated with congruent words, and approach and avoidance temperament scores, computed using a confirmatory factor analysis of several self-report scales, were entered as between-subject predictors. Hemispheric lateralization was tested directly using methods similar to those of Herrington et al. (2010).

Consistent with research on state motivation and regional brain activity, approach temperament moderated activation in two regions of left DLPFC (a relatively anterior region in BA 8 and 9 and a more posterior area in BA 9 only), and avoidance temperament moderated activation in one region of right DLPFC (BA 9 and 6), all of which were lateralized effects. More specifically, higher levels of motivational temperament were associated with greater activation in the condition requiring stronger inhibition (incongruent, relative to congruent). Furthermore, higher levels of temperament were associated with better performance on the task, supporting the important moderating effect of such motivational tendencies on executive function.

The areas of DLPFC observed in Spielberg et al. (2011) have been associated with a number of other functions, including behavioral inhibition, planning action, attending to cues predicting the occurrence of a motivationally salient event, and responding when motivationally salient events occur (Volle et al., 2005; Abler et al., 2006; Bickel et al., 2009; Kaladjian et al., 2009). Incorporating this research with their findings, and consistent with Herrington et al. (2010), Spielberg et al. (2011) hypothesized that these regions of DLPFC are involved in implementing a motivational set that biases lower-order processing (i.e., attention to ink color vs. word meaning) to be congruent with goals. These findings have recently been replicated using an emotion-word Stroop task (Spielberg et al., 2012a), supporting the generalizability of these conclusions.

Although trait (e.g., motivational temperament) and state motivational manipulations are useful individually, it is likely that a deep understanding of the neural mechanisms involved in integrating motivation and executive function will occur only through a combination of these methods (e.g., investigating how trait phenomena moderate state manipulations). This is because a specific outcome can be viewed as an approach and/or avoidance goal depending on the motivational tendencies of each individual. For example, the potential to win a monetary reward will likely be viewed as an approach goal by many individuals, but may also be viewed as an avoidance goal (e.g., an opportunity to perform poorly and miss out on winning). Taking motivational temperament into account will allow for a greater level of certainty regarding the manner in which particular outcomes will be viewed.

### Intertemporal choice

Neuroscience research in the field of intertemporal choice, which investigates choices between outcomes that differ in temporal delay and reward/punishment magnitude, provides another avenue to examine the instantiation of motivational systems in the brain. Humans are often faced with choices between options that differ in the timescale of the potential outcomes. Often, one option is associated with a shorter delay and a smaller reward, whereas the delay in the other option is longer but the reward value greater. For example, an individual may have the goal of losing weight and be faced with the choice of whether to eat a high-calorie desert. In order to maximize gain/minimize loss over time, goals (e.g., losing weight) must be maintained in the face of competing options (e.g., sensory pleasure now), which is a process that can be associated with the system level of the hierarchical model of approach/avoidance. Although the choice with the longer delay has an objectively better outcome, this option is often not chosen, because humans discount the value of delayed rewards (Ainslie, 2001). The rate of future discounting can be thought of as a measure of impulsiveness, because it reflects the tendency to forego larger, long-term rewards in order to gain more immediate satisfaction (Ainslie, 1975). This proposal is supported by research indicating that more impulsive individuals (e.g., children with ADHD) discount future rewards more than less impulsive individuals (Barkley et al., 2001).

Recent research has attempted to identify brain regions involved in integrating temporal delay into the decision-making process. Several studies suggest that DLPFC, medial prefrontal cortex (MPFC), and posterior cingulate cortex (PCC) are involved in decisions to forego proximal reward or incur proximal punishment in order to maximize benefit over time, providing further support for the hypothesis that the system level is instantiated (in part) in DLPFC. Specifically, several studies have found that greater activation in DLPFC, MPFC, and PCC predicted the choice of the larger, later outcome (McClure et al., 2007; Wittmann et al., 2007; Weber and Huettel, 2008; Ballard and Knutson, 2009). As well, activation in DLPFC, MPFC, and PCC has been found to be positively correlated with the length of the delay associated with outcomes (Luhmann et al., 2008; Ballard and Knutson, 2009). Finally, gray-matter volume in DLPFC has been found to be positively associated with the tendency to choose the larger, later outcome over the smaller, more immediate outcome (Bjork et al., 2009).

In these studies, the delay and magnitude associated with each option were explicitly presented to participants. Therefore, maximizing reward over time required only the ability to resist the earlier option. In many real-world choices, however, the magnitude and delay of the outcome will not be explicit. For example, when choosing whether to forego eating (immediately available) cake in order to lose weight, the impact of cake eating on weight and how long it will be until the desired amount of weight will be lost (if cake is not eaten) will usually be unclear. In these situations, learning history can play an important role in determining which choice will be selected (e.g., how quickly a specific individual has lost weight in the past). Several studies have examined intertemporal choice when participants must learn the contingencies associated with different options. For example, Tanaka et al. (2004) employed a decision-making task in which participants had to learn to incur small, immediate losses in order to gain large, delayed rewards. Results revealed that learning to obtain larger, later rewards was associated with increased activation in left DLPFC, MPFC, and PCC, which is consistent with research linking these brain regions to foregoing proximal reward or incurring proximal punishment in order to maximize reward over time.

Yarkoni et al. (2005) employed a task similar to that of Tanaka et al. (2004). However, in their task, the strategy of foregoing more immediate rewards to obtain the delayed reward did not always maximize the total reward over time. Instead, it was optimal to choose the immediate reward in one of the conditions. Results revealed that DLPFC activation was associated with optimum performance (i.e., maximizing total reward) on the task. Specifically, when reward was maximized by sacrificing smaller, earlier rewards to obtain larger, later rewards, sustained activation in DLPFC across the entirety of the trials was greater than activation during the time at which participants actually made choices. When reward was maximized by choosing smaller, earlier rewards, DLPFC exhibited greater activation during the actual choice period, relative to the sustained activation across trials. This indicates that the involvement of DLPFC is not restricted to obtaining delayed rewards. Rather, DLPFC appears to be involved in maximizing overall benefit.

Taken together, this research supports the hypothesis that DLPFC is involved in maximizing benefit/minimizing harm over time. This would involve both the maintenance of appropriate goals in the face of competition (e.g., foregoing a small proximal reward for a larger, delayed reward) and the selection of appropriate strategies (e.g., determining whether obtaining proximal rewards or foregoing proximal rewards for larger, delayed rewards will maximize total benefit over time). Therefore, this research provides evidence that DLPFC is involved in instantiating both the system and strategic levels of the hierarchical model of motivation.

Both MPFC and PCC also appear to play significant roles in maximizing total reward over time. For example, research by Maddock (1999) indicates that PCC is involved in integrating emotional and motivational information with memory during recall. This suggests a role for MPFC and PCC in the anticipation of delayed rewards. When choosing between potential rewards, a representation of each outcome, incorporating motivationally relevant information based on past experience, is needed in order to evaluate the predicted subjective value of the outcome. In addition, the anticipation period itself can have value (Berns et al., 2007), because anticipation can be pleasant or unpleasant (or neutral). Greater incorporation of motivationally relevant information into mental simulations of a potential outcome will make an option seem more or less attractive.

The involvement of MPFC and PCC in the anticipation of potential outcomes is supported by several studies, including a study that found increased MPFC and PCC activation when participants self-reflected on both approach- and avoidance-related goals (Johnson et al., 2006). Additionally, dissociation in PCC activation to motivationally relevant stimuli has been found in relation to approach and avoidance (Touryan et al., 2007). Specifically, when an approach orientation was induced, greater activation in PCC was observed during the evaluation of pleasant stimuli (relative to unpleasant stimuli). In contrast, when an avoidance orientation was induced, greater activation in PCC was observed during the evaluation of unpleasant stimuli (relative to pleasant stimuli). Finally, Peters and Buchel (2010) directly investigated the impact of imagery associated with potential future outcomes on temporal discounting. Participants performed a classic delay discounting task in which they chose between immediate and delayed rewards. Before performing the task, participants identified a number of planned future events (e.g., going to a workshop, going to a friend’s wedding). In one condition, the delayed reward choice was linked to one of the identified future events (i.e., the reward would be given on the day that the event occurred). Results revealed that rewards were discounted less heavily in this condition, relative to a control condition in which no links to future events were presented. Additionally, vividness ratings of future events correlated negatively with the rate of discounting, such that greater vividness was associated with less discounting. Importantly, both MPFC and PCC exhibited greater activation when links were presented, relative to the control condition, suggesting that these regions are involved in the representation of goals via imagery or other means. As well, the subjective value of the delayed reward option (i.e., the objective value multiplied by the delay discount rate) was correlated with brain activation in MPFC and PCC during the condition in which links to future events were presented, suggesting that these regions are involved in representing the value of future outcomes through associated imagery.

These findings are consistent with a model of the neural instantiation of prospection (Buckner and Carroll, 2007). Prospection is the process by which past memories are used to envision potential future scenarios, and this process can be used to assist in planning for future goals. Buckner and Carroll (2007) suggested that MPFC and PCC, along with other areas, are vital to the process of prospection.

In summary, the present review of the literature on intertemporal choice supports the hypothesis that DLPFC plays an essential role in goal pursuit and additionally implicates MPFC and PCC as being important components due to their involvement in the representation of motivationally salient aspects of potential future outcomes. In combination with the research reviewed above on the interaction of motivation and executive function, this research provides a starting point for a model of motivation in the brain.

## A Model of Approach/Avoidance Goal-Pursuit Processes in the Brain

Converging lines of research suggest that DLPFC implements a motivational set that biases lower-order neural processes to facilitate the achievement of goals. It is proposed here that this research can be interpreted by applying the framework of the hierarchical model of motivation (Elliot, 2006; Scholer and Higgins, 2008) to a set of proposals (for reviews, see Botvinick, 2008; Badre and D’Esposito, 2009) that superior, lateral prefrontal cortex (SLPFC), including DLPFC, is organized along a dimension of abstraction. Generally, more anterior regions (e.g., BA 10, DLPFC) are involved in the most abstract aspects of goal-directed processing (e.g., maintaining the ultimate goal), and more posterior regions (e.g., pre-motor cortex) are involved in processing the least abstract aspects (e.g., planning motor sequences).

There is some disagreement regarding the nature of the abstraction that organizes SLPFC. One proposal is that the abstraction is temporal in nature. Specifically, goals become more abstract as the timescale of the task they direct increases (Badre and D’Esposito, 2009). According to Botvinick (2008), timescale is likely the key parameter that governs the organization of SLPFC. Specifically, more anterior regions guide behavior over a longer time-span than do more posterior regions. Another proposal is policy abstraction, in which more abstract goal representations are more general than lower-level goal representations (Badre and D’Esposito, 2009).

**Figure 2:**
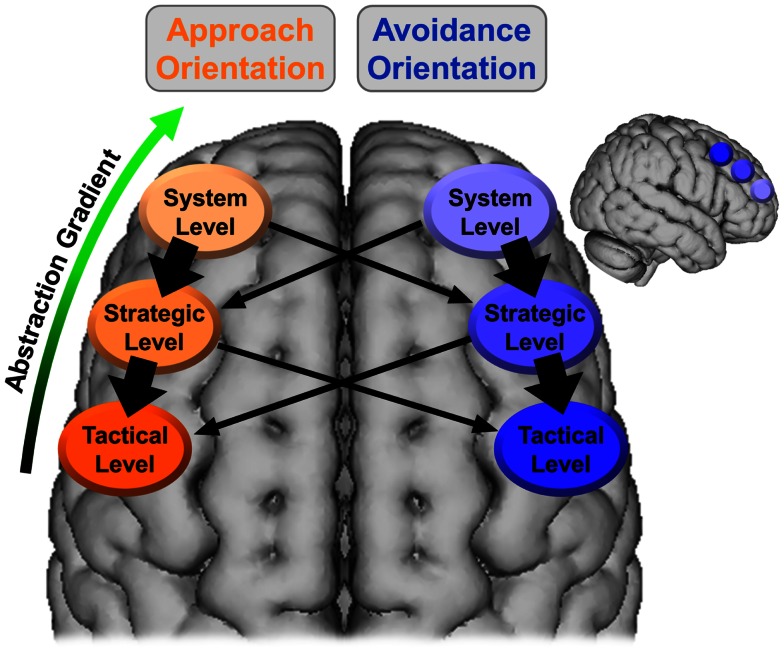
**Lateralized organization of superior, lateral prefrontal cortex with regard to the hierarchical model of motivation**. The thickness of the arrows corresponds to the hypothesized strength of the relationship. The larger brain is an axial view of the superior surface of the brain viewed from above. The smaller brain is a sagittal view of the lateral surface of the right hemisphere. The location and coverage of the ovals/circles is meant to represent a relative placement rather than a delineation of specific cortex.

It is proposed here that the gradient of abstraction and timescale evident in the hierarchical model of motivation can be mapped onto this SLPFC gradient, with the system level associated with more anterior SLPFC (e.g., BA 10, DLPFC) and lower levels (i.e., strategic, tactical) moving sequentially more posterior (e.g., BA 8, pre-motor cortex). The temperament level would be associated with the activity/reactivity of these regions (especially those instantiating the system level) rather than being associated with a specific region of SLPFC. Additionally, it is proposed here that SLPFC is lateralized with respect to motivational orientation, with left SLPFC associated with approach and right SLPFC associated with avoidance. This organization is illustrated in Figure 2. As shown, approach at the system level can recruit both approach and avoidance at the strategic level. However, approach at the system level is more likely to recruit approach at the strategic level, as indicated by the thicker arrows.

Support for this proposal can be found in a study by Kouneiher et al. (2009), which examined the integration of motivation and cognition in the context of a model of PFC abstraction proposed by Koechlin and colleagues (Koechlin et al., 2003; Koechlin and Summerfield, 2007). In this model, posterior DLPFC is hypothesized to be involved in contextual control (i.e., control based on rules related to the immediate context), whereas anterior DLPFC is hypothesized to be involved in episodic control (i.e., control based on a past event which indicates that a certain set of rules should be applied in the current context). The most anterior region, frontopolar PFC (e.g., BA 10), is hypothesized to be involved in branching control (i.e., maintaining a task set in memory while another task is carried out).

Kouneiher et al. (2009) found that rewards/punishments associated with the context (the current trial) and the episode (the current set of trials, which were preceded by a cue signaling the possible incentives) moderated posterior and anterior DLPFC activation, respectively. They also found an anterior-to-posterior gradient in medial PFC, with dorsal anterior cingulate cortex (dACC) activation moderated by episodic motivation and pre-supplementary motor area activation moderated by contextual motivation.

Support for the present proposal that the hierarchical model of approach/avoidance motivation can be mapped onto an anterior/posterior gradient of SLPFC can also be found in a recent study investigating the effect of forming implementation intentions on neural activation associated with goal pursuit (Gilbert et al., 2009). The task contained two conditions, which differed only in whether implementation intentions were externally provided to participants by experimenters. Given that the formation of implementation intentions reduces the need for active engagement of the strategic level (discussed above), and given that the conditions were of equal difficulty and potential monetary reward level, the conditions differed only on the extent to which the strategic level was actively engaged during the task. Consistent with the present proposal, engagement of the strategic level (i.e., when participants were not provided with implementations intentions) was positively associated with activation in two areas of left anterior SLPFC (BA 8 and 10). When participants were provided with implementation intentions, the only area of SLFPC exhibiting differential activation was left pre-motor cortex (BA 6).

### Orbitofrontal cortex

In addition to SLPFC, several other areas are likely to be important components of a model of motivation in the brain. As discussed above, MPFC and PCC appear to play important roles in anticipatory processes. Another potential region is orbitofrontal cortex (OFC), which has been linked to the maintenance of the current and expected motivational value of stimuli (O’Doherty and Dolan, 2006). This area likely provides information about stimulus value to superior areas such as DLPFC (Szatkowska et al., 2008).

However, there is some question regarding the nature of the value representations maintained in OFC (e.g., Roesch et al., 2007). Recent work by Schoenbaum and colleagues (Schoenbaum and Esber, 2010; Jones et al., 2012) suggests that OFC does not maintain cached value representations of a specific stimulus, *per se*. Rather, OFC appears to be necessary for calculating value on the fly in (at least somewhat) novel situations requiring integration of value information from several sources. In other words, OFC is required when an organism must infer the value of a stimulus – not based on direct past experience of the particular situation that is currently faced, but by integrating contingency and value information from different sources to estimate value. This view is supported by research indicating that Pavlovian or simple instrumental conditioning is not impacted by lesioning of OFC (this is not the case for amygdala lesions; Schoenbaum and Esber, 2010). Rather, OFC lesions prevent the suppression of old value when the current context signals a change in value (but no impact is observed after lesioning amygdala). Thus, OFC appears to be more involved in the types of value representations needed specifically in goal-directed behavior, as opposed to habit.

In addition, several studies suggest that the values maintained in OFC are relative to the individual’s current state, rather than to potential future states involved in longer-term goals (e.g., Schoenbaum et al., 1998). For example, when an organism consumes a desired food to satiation (i.e., the food is no longer appealing), OFC tracks stimulus value relative to the current state of the individual (e.g., high value at the start, low or negative value at the end), rather than the long-term value of the food (which should remain high, otherwise that food would not be consumed in the future; Pickens et al., 2003). Additionally, several studies indicate that OFC is associated with choosing smaller, earlier rewards in intertemporal choice paradigms (e.g., Tanaka et al., 2004; Bjork et al., 2009). Therefore, it may be that OFC is involved in value maintenance for (usually shorter-term) goals related to the individual’s current state (e.g., am I hungry now?). For (usually longer-term) goals related to potential future states of the individual (e.g., will I be hungry in the future?), MPFC and PCC may play a similar function by integrating motivationally salient information into memory that is recalled during anticipation. Alternatively, it is possible that OFC is involved in estimating stimulus value based on any state, whether that is the current state or an estimated future state. If so, MPFC/PCC may be more involved in the creation of the potential future state (e.g., estimating future needs/desires in a given circumstance). Future research separating estimation of value and internal state will be valuable in teasing apart these competing theories.

Research indicates that, like SLPFC, OFC may be organized along a gradient of abstraction. Specifically, a recent meta-analysis indicates that posterior OFC is closely associated with more primary reinforcers (e.g., sweet taste), whereas anterior OFC is closely associated with more abstract/secondary reinforcers (e.g., money, pride; Kringelbach and Rolls, 2004). Additionally, O’Doherty and Dolan (2006) have suggested that anterior, medial OFC is associated with maintaining a common neural currency, allowing the values of different types of reinforcers to be compared.

A medial vs. lateral distinction in OFC has been proposed by O’Doherty (2007). Specifically, medial OFC is thought to represent the value of rewards, whereas lateral OFC is thought to represent the value of punishments. However, there appears to be some disagreement regarding the role of lateral OFC. Specifically, Elliott et al. (2000) suggested that lateral OFC is activated when previously rewarded behavior must be inhibited, rather than representing the value of punishments *per se*. Kringelbach and Rolls (2004) incorporated both views and suggested that lateral OFC represents the value of punishments and signals that behavior should change.

This organization of OFC conflicts with the proposal that left PFC is associated with pleasant valence and approach motivation and right PFC with unpleasant valence and avoidance motivation (Heller, 1993; Davidson and Irwin, 1999). Additionally, recent meta-analysis suggests that OFC is lateralized with respect to emotional valence, although not in the predicted direction (Wager et al., 2008). Specifically, bilateral medial and right lateral OFC was associated with pleasant emotional experience, whereas left middle and lateral OFC was associated with unpleasant emotional experience. The association between bilateral, medial OFC and pleasant valence is consistent with O’Doherty’s (2007) proposal. However, the findings of this meta-analysis raise questions regarding the role of lateral OFC that should be pursued in future research.

### Anterior cingulate cortex

Anterior cingulate cortex (ACC) is likely to be another important component of a model of motivation. One theory of ACC function is that ACC is involved in encoding the predicted value associated with actions (for a review, see Rushworth and Behrens, 2008). This includes the immediate reward or punishment value, as well as the value of potential information about future events prompted by the action. Additionally, ACC is hypothesized to influence the degree to which information gained from current actions influences future decisions (Rushworth and Behrens, 2008). Information represented in ACC is needed to efficiently create action plans to pursue goals, suggesting that ACC provides this information to SLPFC, including DLPFC. In relation to the hierarchical model of motivation, information held in ACC will be particularly relevant at the strategic and tactical levels.

An important consideration is to determine the regions of ACC that provide this information, given that several parcellations of ACC have been proposed. One influential parcellation (Bush et al., 2000; Mohanty et al., 2007) divided ACC into two sections; dorsal ACC was hypothesized to be more involved in putatively cognitive tasks such as error processing, whereas rostral ACC was hypothesized to be more involved in putatively emotional tasks. However, mounting evidence has called this parcellation into question. For example, Shackman et al. (2011) supported the association between dorsal ACC and putative cognitive control tasks but found that putatively emotional tasks were equally likely to activate dorsal or rostral ACC. In addition, they found that tasks involving pain were also more likely to activate dorsal ACC. In order to resolve this apparent discrepancy, they proposed that dorsal ACC is involved in using negative consequences (e.g., negative affect, pain) to adaptively modulate behavior, consistent with the proposal by Rushworth and Behrens (2008).

This proposal is also supported by a study that employed both diffusion tractography, which estimates the degree of white matter connectivity with other brain regions, and a meta-analysis of fMRI studies to parcellate cingulate (Beckmann et al., 2009). This analysis identified a region (roughly corresponding to the dorsal ACC region identified by Bush et al. (2000) but extending around the genu of the corpus callosum into rostral ACC) that is heavily connected to DLPFC and surrounding cortex and is reliably activated by reward manipulations. Given that this ACC region displays both motivation-related activation and connectivity to DLPFC, it is likely that this region provides motivational information regarding actions to DLPFC.

### The proposed model

**Figure 3:**
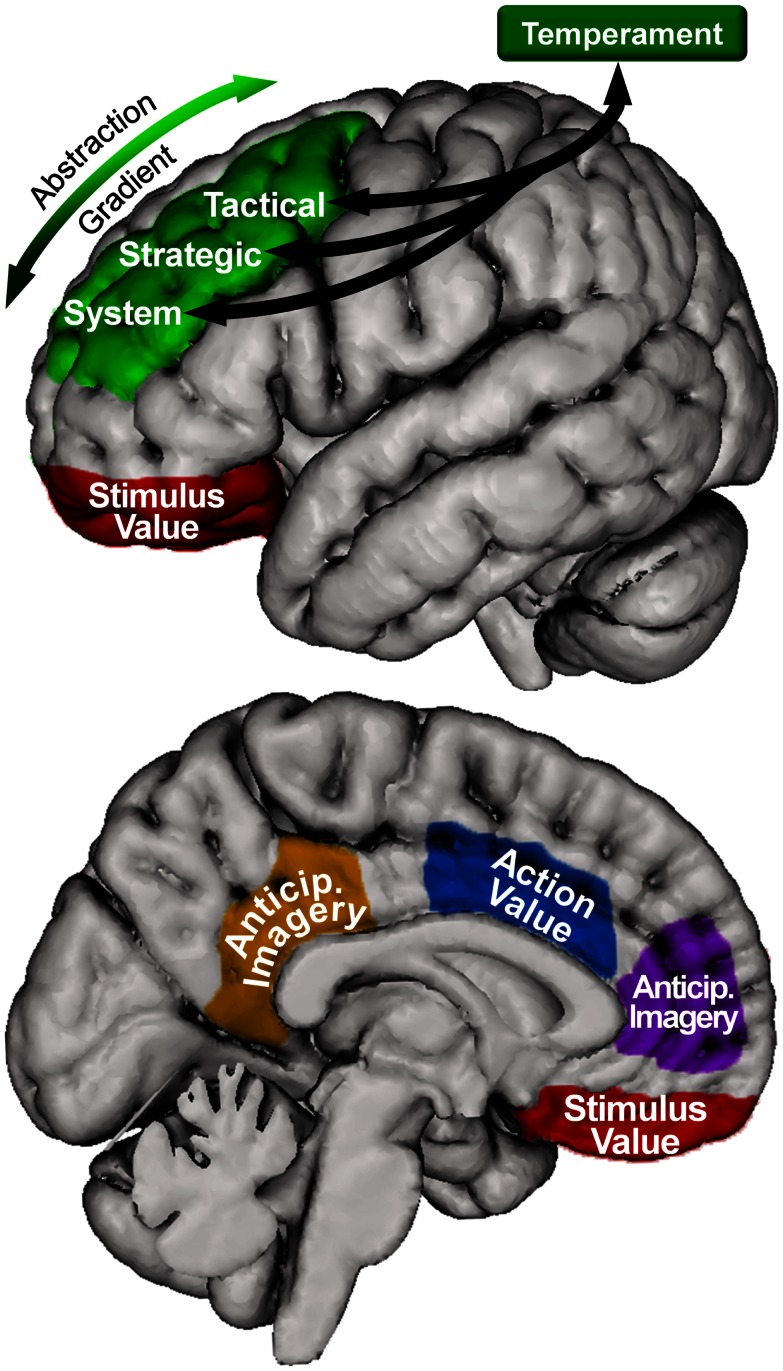
**Motivational organization of superior, lateral prefrontal cortex, and relationship with other brain areas in the model**. Green = superior, lateral prefrontal cortex. Blue = anterior cingulate cortex. Red = orbitofrontal cortex. Purple = medial prefrontal cortex. Orange = posterior cingulate cortex. Only left hemisphere is shown; right hemisphere is similarly organized. The location and coverage of the areas is meant to represent a relative placement rather than a delineation of specific cortex.

The model proposed here (illustrated in Figure 3) posits that the system, strategic, and tactical levels of the hierarchical model of approach/avoidance motivation are instantiated along an anterior-to-posterior gradient of SLPFC (including DLFPC). Further, the present review suggests that OFC and ACC provide information about stimulus and action value, respectively, to these areas. Lastly, MPFC and PCC are involved in integrating motivationally salient information into the anticipation of potential future outcomes. Thus, we propose that all regions discussed are involved in instantiating the model, with SLPFC involved in overall coordination and maintenance over time of level-specific processing.

As an example of how the model may work with a specific goal, an individual may have the approach goal of losing weight in order to be more healthy, which would be maintained in anterior, left SLPFC (e.g., BA 10, anterior DLPFC). In order to pursue this goal, an area of left SLPFC posterior to this (e.g., posterior DLPFC) would be involved in the selection of an approach strategy and would engage ACC in order to obtain information regarding the potential value of different strategies. In this example, two approach strategies could be exercising regularly and eating healthy foods. The healthy eating strategy could be low value/high cost if the individual frequently encounters high-calorie food and has not been successful in the past in losing weight by eating healthily. In contrast, the exercise strategy could be high value/low cost if the individual has easy access to exercise equipment and has been successful in losing weight with exercise in the past, and this would likely be the strategy chosen. Anterior cingulate would also be engaged by a more posterior region of left SLPFC (e.g., pre-motor cortex) in order to determine the value of different approach tactics when judging which tactic to employ. For example, if the strategy were to exercise, an approach tactic could be going to the gym to participate in an exercise class or calling a friend to jog with. If the individual is embarrassed about showing their fitness level in front of strangers, the gym class tactic could be low value/high cost, whereas the tactic of jogging with a friend could be high value/low cost if the friend is sympathetic because they are also out of shape and attempting to lose weight, and this would likely be the tactic chosen. Finally, when faced with a conflicting goal, for example to enjoy a high-calorie dessert, anterior SLPFC would engage MPFC and PCC in anticipatory imagery of the future state of being thin. OFC would be involved in calculating the value of the dessert relative to the current state (though it may also be involved in calculating future value based on the anticipated future state of being thin).

Although the brain areas currently integrated into the model are proposed to be fundamental to the pursuit of goals, they are almost certainly not the only brain areas involved. Other brain regions are likely involved in instantiating fundamental components of motivation and are not yet incorporated into the present model. In addition, specific situations will necessitate the engagement of brain areas that instantiate processes more specific to the demands of that situation. For example, although engagement of Broca’s area is not necessarily fundamental to goal-pursuit generally, it may be vital in situations where verbal rehearsal is needed to complete the task.

### Relationship to extant models

The proposed model overlaps in a number of important dimensions with extant neural models of goal pursuit (although the models discussed are not necessarily labeled as such). For example, a number of models (e.g., Passingham and Wise, 2012) share a hierarchical structure (see Pessoa, 2009, for an example of a less hierarchical model), and this hierarchy is also mapped, at least to some degree, along an anterior-posterior gradient in PFC in some models (e.g., Fuster, 2008). In addition, the regions included in the proposed model are largely common across models (e.g., ACC, OFC).

The present model extends these models in a number of ways. First, the present model considers goal-pursuit related to approach and avoidance motivation separately, a division supported by a long line of research, as discussed above. In addition, the present model incorporates hemispheric asymmetry and the differential mapping of approach and avoidance therein. Furthermore, although previous models share a hierarchical structure, the present model incorporates well-researched psychological models of approach and avoidance to flesh out the nature of this hierarchy.

## Preliminary Tests of the model

A recent study provided preliminary support for the proposed network model (Spielberg et al., 2012b). In two independent samples, this study found that OFC, ACC, MPFC, and PCC interacted with regions of left and right DLPFC associated with approach and avoidance, respectively, to maintain difficult goals. Notably, approach and avoidance motivational temperament were associated with greater connectivity between OFC and left and right DLPFC, respectively, indicating that connections between DLPFC and OFC are of particular importance for motivational processes.

A recent explosion of methods for network identification and description will allow for further testing and refinement of the proposed model. Prominent among these methods is graph theory, which can characterize the role of different brain regions (nodes) within a network (see Bullmore and Sporns, 2009). For example, graph theory can identify that nodes are more central to a network (hubs) by examining the paths by which different nodes communicate with each other. Hubs are those nodes which are more often intermediaries through which different nodes communicate. Graph theory can also isolate specific circuits within the larger network (modules) by identifying nodes that tend to communicate more with nodes within the module than those outside. Importantly, graph theory can be used to test the importance of a node in a network by removing the node and assessing whether the network continues to function efficiently.

Use of graph theory has already begun to bear fruit. For example, Kinnison et al. (2012) examined brain networks during a task involving both motivational and executive function components. Results revealed that reward increased the efficiency of connections, especially between cortical and subcortical regions. With regard to the model proposed here, graph theory can be used to test a number of important components. For example, regions of superior-lateral PFC (e.g., DLPFC) are posited to be central to the pursuit of goals. Therefore, regions such as DLPFC should function as hubs when individuals are pursuing goals. In addition, we expect the regions posited to be nodes in the model to function as a module within the larger suite of brain regions active during goal pursuit. Finally, in combination with tasks that isolate specific aspects of the model (e.g., selection and implementation of tactics), graph theory can be used to test the importance of a particular node (e.g., dACC) to the isolated model aspect. In summary, graph theory, among other methods assessing networks (e.g., Astolfi et al., 2007; Popov et al., 2013), will likely be of great utility in testing and refining the proposed model.

## Model Implications

The proposed model has a number of implications for future research. For example, the type of task manipulation used in a study (i.e., approach vs. avoidance) should be carefully considered, because this information should guide hypotheses about which hemisphere is primarily involved. If a task manipulation could be both approach- and avoidance-related (e.g., across participants), the power of the experiment may be diluted, because some participants primarily engage left SLPFC, whereas others engage right. Another implication is that when conducting research aimed at understanding goal pursuit or control processes in the brain, researchers should be aware of the motivational level(s) (e.g., system vs. tactical) manipulated by their task and examine specific areas of SLPFC (e.g., anterior DLPFC vs. pre-motor cortex) accordingly. If a task manipulation engages different levels at different times, and this is not accounted for in the analysis strategy, power may also be reduced, because SLPFC regions will not be consistently activated during the manipulation. Alternatively, if a task manipulation simultaneously engages multiple levels, specificity regarding regions of SLPFC involved may be lost. Another consideration implicated by the present model is the time frame in which goal manipulations operate (e.g., relative to a current or future state), which should be examined to determine whether value information is likely to be represented in OFC and/or MPFC/PCC.

The present model encourages the use of neuroscience data to drive psychological research on goal-pursuit processes. For example, novel hypotheses can be generated based on what is known about the brain regions involved in implementing those processes. Additionally, the present model is more spatially specific than previous models and provides an initial structure for the interactions among regions. This can be used, for example, to guide the placement of sources in EEG and MEG source localization research. This allows both for disentangling activity related to multiple, simultaneously occurring goal-pursuit processes associated with different brain areas in the model and for examination of the interactions among regions with great temporal specificity (see Silton et al., 2010, 2011, for examples of the utility of this technique). Finally, the present model can improve the utility of neuroscience research on goal-pursuit and control processes by providing a framework, incorporating a rich psychological conceptualization of approach/avoidance motivation, in which to place this research.

Reflected in the present model is an attempt to draw on nuanced psychological conceptualizations of approach/avoidance motivation (e.g., Elliot, 2006; Scholer and Higgins, 2008) to provide a more specific model (in terms of both brain regions and psychological processes involved) of how motivation is instantiated in brain networks. This model benefits from being informed by several often disconnected literatures, including psychological and neuroscience research on the structure of approach/avoidance approach/avoidance goal pursuit.

## Conflict of Interest Statement

The authors declare that the research was conducted in the absence of any commercial or financial relationships that could be construed as a potential conflict of interest.
